# Amino Acid Formula Containing Synbiotics in Infants with Cow’s Milk Protein Allergy: A Systematic Review and Meta-Analysis

**DOI:** 10.3390/nu13030935

**Published:** 2021-03-14

**Authors:** Katy Sorensen, Abbie L. Cawood, Glenn R. Gibson, Lisa H. Cooke, Rebecca J. Stratton

**Affiliations:** 1 Medical Affairs, Nutricia Ltd., White Horse Business Park, Trowbridge BA14 0XQ, UK; 2 Institute of Human Nutrition, Faculty of Medicine, Mailpoint 113, Southampton General Hospital, Tremona Road, Southampton SO16 6YD, UK; a.l.cawood@soton.ac.uk (A.L.C.); r.j.stratton@soton.ac.uk (R.J.S.); 3 Department of Food and Nutritional Sciences, University of Reading, Whiteknights, Reading RG6 6AP, UK; g.r.gibson@reading.ac.uk; 4 Department of Nutrition and Dietetics, Bristol Royal Hospital for Children, Upper Maudlin Street, Bristol BS2 8BJ, UK; lisa.cooke@uhbw.nhs.uk

**Keywords:** paediatrics, allergy, cow’s milk protein allergy, synbiotics, gut microbiome, amino acid formula, clinical outcomes

## Abstract

Cow’s milk protein allergy (CMPA) is associated with dysbiosis of the infant gut microbiome, with allergic and immune development implications. Studies show benefits of combining synbiotics with hypoallergenic formulae, although evidence has never been systematically examined. This review identified seven publications of four randomised controlled trials comparing an amino acid formula (AAF) with an AAF containing synbiotics (AAF-Syn) in infants with CMPA (mean age 8.6 months; 68% male, mean intervention 27.3 weeks, *n* = 410). AAF and AAF-Syn were equally effective in managing allergic symptoms and promoting normal growth. Compared to AAF, significantly fewer infants fed AAF-Syn had infections (OR 0.35 (95% CI 0.19–0.67), *p* = 0.001). Overall medication use, including antibacterials and antifectives, was lower among infants fed AAF-Syn. Significantly fewer infants had hospital admissions with AAF-Syn compared to AAF (8.8% vs. 20.2%, *p* = 0.036; 56% reduction), leading to potential cost savings per infant of £164.05–£338.77. AAF-Syn was associated with increased bifidobacteria (difference in means 31.75, 95% CI 26.04–37.45, *p* < 0.0001); reduced *Eubacterium rectale* and *Clostridium coccoides* (difference in means −19.06, 95% CI −23.15 to −14.97, *p* < 0.0001); and reduced microbial diversity (*p* < 0.05), similar to that described in healthy breastfed infants, and may be associated with the improved clinical outcomes described. This review provides evidence that suggests combining synbiotics with AAF produces clinical benefits with potential economic implications.

## 1. Introduction

Cow’s milk protein allergy (CMPA) is one of the most common food allergies in children, typically diagnosed in the first year of life with an estimated prevalence of around 2–5% of infants in Europe [1,2,3,4]. Analysis of National Health Service (NHS) records showed that, overall, allergic conditions account for 12.5 million GP consultations and 183,000 admitted bed days annually in the UK, costing over £1bn [5], with additional direct and indirect household costs [6]. Moreover, the impact of CMPA on quality of life is substantial, with anxiety, frustration and social limitation being prominent issues for families [7,8]. Around 44% of infants with CMPA show a specific IgE response, with the remainder of cases being considered non-IgE mediated [4]. However, the presence or absence of specific IgE alone should not determine treatment, as approaches to management are based on eliminating exposures to cow’s milk protein (CMP) and may vary according to age of the infant, severity of the condition and current feeding methods [2,3,9].

Breastfeeding remains the best option in CMPA and generally avoids exposure to CMP. Where breastfeeding is not possible, partially or fully formula-fed infants with CMPA may require management with hypoallergenic formulae (HAF) [2,3,9]. Most guidelines recommend the use of extensively hydrolysed formula (eHF) first-line in the majority of infants with CMPA; in severe or complex CMPA, or where symptoms do not resolve with eHF, amino acid formula (AAF) is recommended [2,3,9,10]. Although HAF are recommended in clinical guidelines, impacts on gut microbiota are important considerations, as they are widely recognised to play an important role in allergy and immune development [11,12,13].

In healthy breastfed infants, faecal microbiota is characterised by a relative preponderance of *Lactobacillus* and *Bifidobacterium*, with fewer other organisms such as *Bacteroides*, *Clostridium*, and Enterobacteriaceae [14,15,16,17]. CMPA is associated with gut dysbiosis [14] and the development of other allergic conditions in later childhood [12,13]. Whilst difficult to establish causality, it is hypothesised that early intestinal dysbiosis may disrupt regulatory mechanisms of the immune response, triggering pro-allergic processes and increased risk of allergy [18]. Therefore, modification of the gut microbiome warrants investigation as a potential strategy in CMPA management. One approach to this is the use of prebiotic and probiotic-supplemented infant formulae.

A probiotic consists of live microorganisms intended to improve health [11] while a prebiotic is a microbial substrate that allows specific changes in the composition and activity of the gastrointestinal microbiota to confer a health benefit [19,20]. Probiotics (given within formulae or as an adjuvant treatment to formula) have been associated with earlier resolution of CMPA [21] and the use of formula containing prebiotics has been shown to enhance levels of *Bifidobacterium* species in formula-fed infants [22,23], being more similar to those of healthy breast-fed infants [24]. When used together, probiotics and prebiotics are termed complementary synbiotics [25]. The impact of synbiotics when taken as an adjuvant to both breast-feeding and formula feeding has also been evaluated in infants with CMPA, showing increases in weight and head circumference [26], enhanced gut *Lactobacillus* and *Bifidobacterium* [27] and reductions in atopic eczema [27,28], with lower risk of respiratory infections and antibiotic usage [28]. There is also emerging evidence from individual randomised controlled trials (RCTs) investigating the effect of hypoallergenic formula containing synbiotics, but to date there has not been a comprehensive review summarising these findings. Therefore, this systematic review was undertaken to examine whether HAF (eHF and AAF) containing synbiotics could have a beneficial effect on clinical outcomes, including clinical symptoms and allergenicity, rates of infections, hospitalisation, medication usage, gut microbiota colonisation, stool characteristics and growth in infants with CMPA.

## 2. Materials and Methods

### 2.1. Identification and Selection of Studies for the Systematic Literature Review

An online search strategy, devised in accordance with PRISMA guidelines, was carried out using MEDLINE, EMBASE and Cochrane Library up to November 2020. Specific search strategies were developed for each data source using an initial broad approach based on the following structure: Infant Feed [SUBJ] OR Milk [TW] OR Formula [TW] AND Probiotic [SUBJ/TW] OR Prebiotic [SUBJ/TW] OR Synbiotic [SUBJ/TW]. Hand searching of pre-determined international allergy conference proceedings (EAACI; EAACI PAAM; FAAM-EUROBAT) was undertaken and secondary searches were carried out by hand-searching reference lists of all full text publications obtained. Previously published systematic reviews were particularly sourced for this purpose.

After elimination of duplicates, identified abstracts were screened against defined inclusion and exclusion criteria. Only publications reporting on RCTs qualified for review. Subjects eligible for inclusion were infants and children aged <3 years with IgE or non IgE mediated CMPA. Studies of older children, children without CMPA, adults and animals were excluded. Suitable interventions were any AAF with synbiotics (AAF-Syn) or eHF with synbiotics (eHF-Syn). Studies of infant formulae containing cow’s milk protein, and eHF or AAF without synbiotics (including those with only a prebiotic or a probiotic) were excluded. No restrictions were placed on sample size, duration of intervention, or type of comparator. Full papers, abstracts and conference proceedings were eligible for inclusion. Only English language publications were eligible.

### 2.2. Quality Assessment

Quality assessment was conducted by one researcher and verified by a second assessor. Any discrepancies were resolved by discussion. Publications which passed the initial screen were obtained in full to ascertain a final inclusion decision. The quality of all included publications were assessed using the Cochrane Risk of Bias tool for randomised trials (ROB-2) [29]. This tool evaluates study quality in five domains; judgement can be ‘Low’, or ‘High’ risk of bias, or can express ‘Some concerns’, meaning a publication has concerns in at least one domain but is not at high risk of bias for any domain. Additional information was obtained through personal communication with the author for the included abstracts [30,31]. It was intended that publications at high risk of bias would not be included in the review.

### 2.3. Data Extraction and Outcome Measures

A pre-determined data collection table was designed to capture study characteristics and outcome data. Outcome measures sought included clinical symptoms (e.g., atopic dermatitis, general allergy symptoms), infections and healthcare usage (e.g., medication and hospitalisation), gut microbiota profiles from faecal samples, growth and stool characteristics. Direct communication with publication authors was undertaken where needed to obtain additional data or clarification. Following data extraction from eligible studies, meta-analysis was conducted for comparable data where possible. Outcomes that could not be meta-analysed are described in the text.

### 2.4. Statistical Methods

Throughout this review, data were presented as means from the RCTs (*n* = 4) not from the publications (*n* = 7) to avoid any double counting. To account for differences in sample size between studies, data (including age, intervention duration and intake) were presented as weighted averages and, in some instances, as cumulative averages. Cumulative averages were calculated by pooling data across studies and weighting results according to study sample size, omitting data from one publication [32] where the same outcome was reported in the study follow-up publication [33] to avoid double counting.

Meta-analysis was conducted using Comprehensive Meta Analysis Version 3 (Biostat©, New Jersey, USA), using a fixed effects model, with random effects for sensitivity analysis where the I^2^ approached 50 or over. Data were presented as either difference in means and 95% confidence intervals (95% CI) or odds ratio and 95% CI. Meta-analysis of microbiota data was undertaken using data extracted from the last timepoint of each publication. In the absence of quantifiable data for the whole cohort, subset data (for infants who received the intervention for the full 26 week follow-up) was used for one publication [33]. Sensitivity analysis was conducted using the first post-intervention timepoint for each publication. Where mean values were not available, the median was used, in line with Hozo et al. [34]. If not explicitly stated, standard deviation was estimated by back-calculation from published confidence intervals. Where this was not available, an estimate was made from the median and range values, as described elsewhere [34]. Overall significance was assumed at *p <* 0.05. Forest plots were used to present meta-analysis data. Further details of the statistical methods associated with meta-analysis are described elsewhere [35].

### 2.5. Simple Cost Analysis

A simple cost-analysis was conducted from the data available on hospital admissions [31], based on the average cost of a paediatric hospital admission for major infection [36] and accounting for the cost of HAF containing synbiotics and HAF (without synbiotics) [37], to estimate an annual healthcare budget impact per infant.

## 3. Results

### 3.1. Overall Search Findings (n = 7)

**Figure 1:**
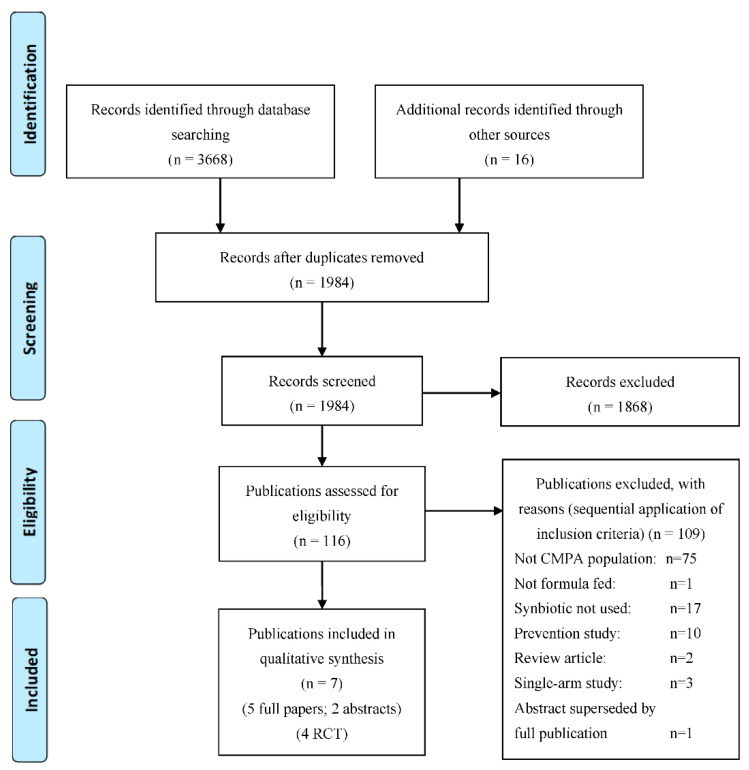
PRISMA flow chart.

A total of 3684 publications were identified by the search strategy (Figure 1). After removal of duplicates, and evaluation of the title and/or abstract, 116 publications were potentially relevant and obtained in full. Following full text review, seven publications (5 full papers, 2 abstracts) were deemed eligible for inclusion, reporting data from 4 RCTs (*n* = 410; mean age 8.6 months; 68% male) [30,31,32,33,38,39,40]. The other 109 publications were excluded from the systematic review due to not including a CMPA population (*n* = 75, of which four were full papers including eHF-Syn in atopic infants); infants not being formula fed (*n* = 1); not including synbiotics (*n* = 17); being a prevention study (*n* = 10); being a review article (*n* = 2); being a single-arm study (*n* = 3 abstracts including eHF-Syn in infants with CMPA); and being an abstract superseded by a full publication (*n* = 1).

### 3.2. Description of Publications Included in the Systematic Review (n = 7)

The seven publications [30,31,32,33,38,39,40] included in the systematic review are shown in Table 1 and Table 2. These report results from 4 RCTs on the use of AAF with synbiotics (AAF-Syn) in infants with CMPA (of which one also reported on a one-arm double blind placebo-controlled crossover food challenge [38]). Two of the full papers [33,40] report additional outcomes from the same primary RCT (ASSIGN study) [32] and both abstracts report different outcomes from the same primary RCT (PRESTO study [30,31]). In all seven publications, infants with confirmed CMPA (including infants with IgE mediated and non IgE mediated CMPA) were given a standard AAF supplemented with the probiotic *Bifidobacterium breve* M16-V and prebiotics (including chicory-derived neutral oligo-fructose and long chain inulin). No eligible publications of alternative HAF with synbiotics were identified by this review. Although the systematic search found four studies [41,42,43,44] of eHF-Syn, they were conducted in atopic infants but not with a diagnosis of CMPA, and were therefore excluded. Intervention periods across the 7 included publications ranged from 7 days to 12 months (weighted mean 4 RCT [31,32,38,39] 27.3 weeks), with follow-up for the included outcomes ranging from 7 days to 12 months. The total number of patients studied in a single RCT ranged from 60 to 169 (Table 1). The average daily intake of the AAF with synbiotics reported ranged from 349 mL to 652 mL (calculated intakes based on standard dilution of 237 to 443 kcal, 6.6 to 12.4 g protein; weighted mean without double counting publications of the same RCT [31,32,38]: 389 mL, 265 kcal, 7.4 g protein).

**Figure 2:**
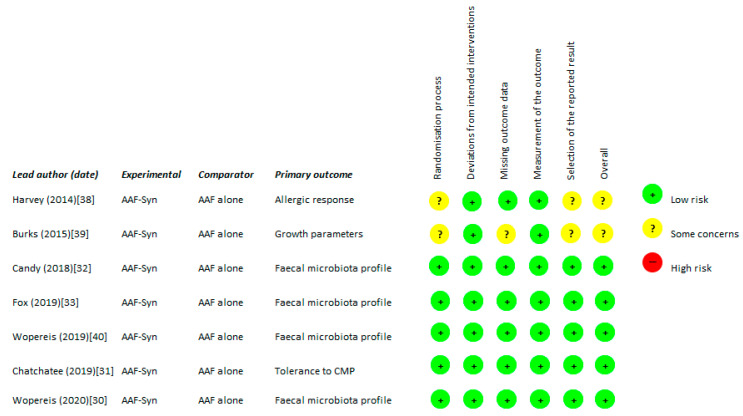
Results of risk of bias assessment for the seven included publications (ROB-2 [29]). Abbreviations: AAF-Syn: amino acid formula with synbiotics. AAF: amino acid formula. CMP: cow’s milk protein.

When assessing the quality of the studies, overall the majority of publications were at low risk of bias (Figure 2).

### 3.3. Outcomes from Studies Comparing AAF with Synbiotics with AAF Alone

#### 3.3.1. Clinical Symptoms & Allergenicity

Four publications (3 RCT; *n* = 241) reported clinical symptoms and allergenicity in infants with IgE or non IgE mediated CMPA who received AAF-Syn or AAF for periods ranging from 7 days to 16 weeks [32,33,38,39], although quantitative data was not given in all cases (Supplementary Table S1). Three publications reported on SCORing Atopic Dermatitis (SCORAD) [45] and on clinical rating scales such as gastrointestinal, skin, respiratory and general symptoms [32,33,39] and one publication reported data from a double-blind placebo-controlled crossover food challenge (DBPCCFC) [38]. Data from all publications showed that both AAF-Syn and AAF were hypoallergenic. Improvement in clinical symptoms were reported over time in both groups, with no significant differences between groups in all three publications reporting symptoms, and both formulae led to a negative response in a DBPCCFC [38].

#### 3.3.2. Infections and Hospital Admissions

Four publications (3 RCT; *n* = 350) reported data on infections from adverse event data, in infants with IgE and non IgE mediated CMPA who received AAF-Syn or AAF alone, over periods from 8 weeks to 12 months (Table 3) [31,32,33,39]. All four publications reported fewer infants receiving AAF-Syn had infections, compared to those receiving AAF alone, which was statistically significant in two publications [31,39]. One of these publications reported additional specific data on ear infections, finding significantly fewer infants who received AAF-Syn had at least one ear infection, compared to those who received AAF alone (0% vs. 20%; *p* = 0.011) [33].

Pooled analysis of infections data from three of the four publications (omitting data from one publication [32] to avoid double-counting) showed a lower cumulative average percentage of infants who had infections with AAF-Syn vs. AAF alone (13.6% vs. 27.8%, respectively) (Table 3), equating to more than 50% reduction overall.

**Figure 3:**
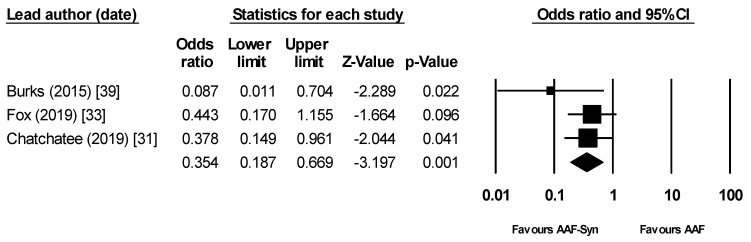
Meta-analysis showing significantly lower rates of infection with AAF-Syn vs. AAF. Abbreviations: AAF-Syn: amino acid formula with synbiotics. AAF: amino acid formula. CI: confidence intervals.

Meta-analysis (omitting data from one publication [32] to avoid double-counting) showed that the proportion of infants who had infections was significantly lower with AAF-Syn than with AAF alone (OR 0.35 (95% CI 0.19 to 0.67), *p* = 0.001, I^2^ = 0, fixed effects, 3 publications, Figure 3) [31,33,39].

Hospital admissions arising from infections were reported in one publication [31], which showed significantly fewer infants had admissions with AAF-Syn compared to AAF alone (8.8% vs. 20.2%; *p* = 0.036), equating to a 56% reduction.

##### Simple Cost Analysis Based on Hospital Admission Data

The approximated average cost of a paediatric hospital admission for a serious infection in England ranges from £1756 to £7792 [36], with a calculated mean cost per patient per admission of £3811. Based on this, looking at hospital admission rates alone, assuming each patient within this review who was recorded as having hospital admissions had only one admission, and accounting for the cost of the AAF-Syn and AAF powders [37], there is an annual potential mean cost saving of between £164.05 per infant (based on reported intakes and duration from the publication which reported hospital admissions [31]) and £338.77 per infant (based on weighted mean intake and duration data from all included RCTs which reported intakes [31,32,38]).

#### 3.3.3. Medication Use

Three publications (2 RCT; *n* = 181) reported data on medication usage [32,33,39] in infants with IgE and non IgE mediated CMPA who received either AAF-Syn or AAF for 8 to 16 weeks (Table 4). Medications reported across the publications included overall concomitant medication use (not specified); antibacterials and anti-infectives (which includes antibiotics); dermatologicals; antifungals; emollients; and functional GI medications [32,33,39].

All three publications which had available data on antibacterial, anti-infective or antibiotic usage found fewer infants in the AAF-Syn group used these medications, compared to the AAF group (range 9–17% with AAF-Syn vs. 31–34% with AAF). This was shown to be statistically significant in two publications (*p* < 0.05) [32,39]; whilst a *p*-value was not available in one publication [33] as data were calculated from details of the subgroups who received systemic antibiotics during the study period. Pooled analysis showed the weighted average percentage of infants using antibacterials, anti-infectives or antibiotics was 15% with AAF-Syn vs. 33% with AAF, equating to a 55% reduction. When omitting one publication [32] to avoid double counting, results were similar (17% vs. 33%, respectively; 48% reduction).

Similarly, all three publications found lower usage of other medications with AAF-Syn than with AAF alone, with statistically significant differences reported in two of the three publications (*p* < 0.05) [33,39]. Of these, one publication reported extensively on ‘other’ medication usage, finding fewer infants using ‘other’ medications with AAF-Syn compared to AAF alone across all categories, most significantly for dermatologicals (*p* = 0.019), emollients and protectives (*p* = 0.023) and antifungals (*p* = 0.054) [33]. One publication reported significantly fewer infants received functional GI medication (*p* = 0.029) [39] with AAF-Syn. Pooled analysis showed that AAF-Syn was associated with a lower overall percentage of infants using emollients, protectives and dermatological medications (−69%); and concomitant medications (−19%; −14% when omitting one publication [32] to avoid double counting).

#### 3.3.4. Change in Gut Microbiota Profile

In total, five publications (3 RCT; *n* = 350) reported on gut microbiota from faecal samples in infants with IgE and non IgE mediated CMPA, following feeding with AAF-Syn or AAF for periods ranging from 8 weeks to 12 months [30,32,33,39,40] (Table 5). The different ways in which this was measured in each publication is summarised below.

Of these five publications that reported on gut microbiota from faecal samples, three provided data on faecal proportions of bacteria (*Bifidobacterium* species and *Clostridium histolyticum* (CH), *Eubacterium rectale* (ER) and *Clostridium coccoides* (CC) groups) as a percentage of total bacteria, from baseline to timepoints ranging from 8 to 26 weeks [32,33,39]. Of these, two publications reported data from a subgroup analysis of infants who a) had not received antibiotic treatment and b) had remained on the originally allocated formula until week 26 (completers) [32,33].

One publication reported data on faecal bacterial diversity (phylogenetic diversity (PD) and Shannon index (SI)) assessed by 16S rRNA-gene sequencing [40]. One publication did not report detailed data, but described differences in relative abundances of *Bifidobacterium* species, *Lactobacillus* species, *Blautia*, *Tyzzerella* and *Romboutsia*, and differences in faecal bacterial diversity, at 6 and 12 months [30].

Two publications reported data on markers of bacterial metabolic activity including pH, short chain fatty acids and lactic acids [39,40]. Two publications also provided comparative data from a reference group of 51 age-matched healthy breastfed infants on faecal proportions of bacterial species [32], faecal bacterial diversity and markers of bacterial metabolic activity [40], at 8 weeks.

Data from all three publications [32,33,39] reporting data on faecal proportions of bacteria found that, compared to AAF alone, AAF-Syn resulted in significantly greater percentages of *Bifidobacterium* species and significantly lower percentages of *Eubacterium rectale* and *Clostridium coccoides* species. The fourth publication [30] described similar trends, although statistical significance was not reported. By pooling the available quantifiable data from all post-intervention timepoints of the three publications, a weighted average could be calculated, which showed that the average percentage of *Bifidobacterium* species was higher in infants who received AAF-Syn (44.0%) than those who received AAF alone (12.2%), whilst *Eubacterium rectale* and *Clostridium coccoides* was lower (11.7% vs. 29.5%, respectively). All three publications reported no significant differences between groups at baseline, yet only two provided quantifiable data, which showed that the absolute increase over time in *Bifidobacterium* species was greater with AAF-Syn than AAF alone when taken for 16 weeks (+36.9% vs. −3.7%) [39] and 26 weeks (+22.2% vs. −2.6%) [33]. In addition, they showed a decrease in *Eubacterium rectale* and *Clostridium coccoides* (AAF-Syn −10.0% vs. AAF alone +15.4% over 16 weeks [39]; AAF-Syn −10.5% vs. AAF alone +20.5% over 26 weeks [33]). The higher proportions of *Bifidobacterium* species and lower proportions of *Eubacterium rectale* and *Clostridium coccoides* after feeding with AAF-Syn were found to be more similar to the bacterial profile of healthy breastfed infants (*Bifidobacterium* species 55%, *Eubacterium rectale* and *Clostridium coccoides* 6.5%) by the publication which provided comparative data on faecal bacterial proportions from a healthy breastfed reference group [32].

**Figure 4:**
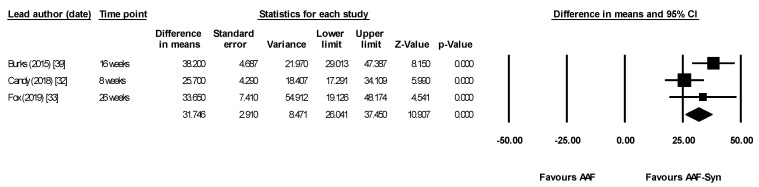
Meta-analysis showing significantly higher percentages of faecal bifidobacterial species with AAF-Syn. Abbreviations: AAF-Syn: amino acid formula with synbiotics. AAF: amino acid formula. CI: confidence intervals.

**Figure 5:**
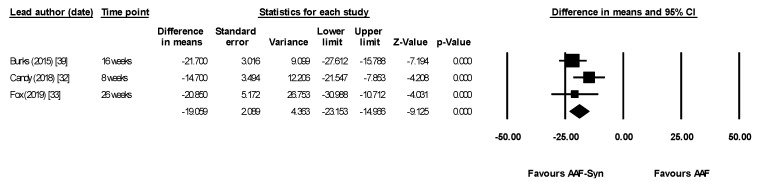
Meta-analysis showing significantly lower percentages of adult-like *Eubacterium rectale* and *Clostridium coccoides* species with AAF-Syn. Abbreviations: AAF-Syn: amino acid formula with synbiotics. AAF: amino acid formula. CI: confidence intervals.

Meta-analysis using data from the last timepoint of each publication (8–26 weeks) showed that AAF-Syn significantly increased percentages of *Bifidobacterium* species (difference in means 31.75, 95% CI 26.04–37.45, *p* < 0.0001, I^2^ = 49.34, fixed effects, 3 publications, Figure 4) and significantly decreased percentages of *Eubacterium rectale* and *Clostridium coccoides* (difference in means −19.06, 95% CI −23.15 to −14.97, *p* < 0.0001, I^2^ = 18.14, fixed effects, 3 publications, Figure 5). When meta-analysis was undertaken using a random effects model for completeness, results were similar and remained significant (*Bifidobacterium* species difference in means 32.15, 95% CI 23.78–40.51, *p* < 0.0001; *Eubacterium rectale* and *Clostridium coccoides* difference in means −19.01, 95% CI −23.63 to −14.40, *p* < 0.0001).

Sensitivity analysis using the earliest post-intervention timepoints of each publication (4–8 weeks) showed similar results (difference in means for *Bifidobacterium* species 28.02, 95% CI 22.01–34.03, *p* < 0.0001, fixed effects, I^2^ = 0, 3 publications; difference in means for *Eubacterium rectale* and *Clostridium coccoides* −14.72, 95% CI −19.17 to −10.27, *p* < 0.0001, fixed effects, I^2^ = 0, 3 publications). When excluding one publication [32] to avoid any potential double counting of the same outcome which was also reported in the study’s follow-up publication [33], the results were similar and remained statistically significant.

Data from the two publications [32,33] that reported subgroup analysis indicated that infants who had not received antibiotic treatment and who remained on their allocated formula for 26 weeks had results consistent with the primary analysis.

Both publications which reported on bacterial diversity showed that this was lower overall with AAF-Syn compared to AAF alone [30,40]. The one publication [40] which reported specific data on bacterial diversity found that, while diversity in faecal microbiota increased over time in both groups, this was less pronounced among infants who received AAF-Syn than those who received AAF alone. At week 8, the bacterial diversity of infants who received AAF-Syn (PD 4.89 ± 1.05; SI 3.75 ± 0.67) was closer to that of the healthy breastfed reference group (PD 4.37 ± 1.14; SI 3.63 ± 0.80) than the infants who received AAF alone (PD 5.17 ± 0.88; SI 4.01 ± 0.71).

The two publications [39,40] that reported data on markers of bacterial metabolic activity showed that, compared to AAF alone, AAF-Syn was associated with significant differences, notably lower faecal pH (5.82 ± 0.72 vs. 6.72 ± 0.47, *p* < 0.001) and propionic acid (11.4% ± 7.3 vs. 22.6% ± 21.4, *p* = 0.006) and higher acetic acid (80.7% ± 10.7 vs. 68.6% ± 21.5, *p* = 0.004) when given for 16 weeks [39], although significant differences were not observed at 8 weeks [40].

#### 3.3.5. Other Outcomes

##### Stool Characteristics

Three publications (2 RCT; *n* = 181) reported stool characteristics (colour, frequency, consistency) in infants with IgE or non IgE mediated CMPA who received AAF-Syn or AAF for 8 to 16 weeks [32,33,39], although quantitative data were not given in all cases (Supplementary Table S2). One publication reported that stool colour was significantly preferred by parents of infants who received AAF-Syn compared to parents of those who received AAF alone, at weeks 0–2 (*p =* 0.014), 2–4 (*p =* 0.010) and 4–12 (*p =* 0.008) [39], while the other two publications reported no significant differences [32,33]. One publication reported slightly lower average stool frequency with AAF-Syn compared to AAF alone at week 8 (1.88 ± 0.19 vs. 1.98 ± 0.15, *p =* 0.015) [32], while two publications found no significant differences between groups [33,39]. No significant differences in stool consistency were reported.

##### Growth

Three publications (2 RCT; *n* = 181) reported growth parameters (weight, length and head circumference) for infants with IgE or non IgE mediated CMPA who received AAF-Syn or AAF for periods ranging from 8 to 16 weeks [32,33,39] (Supplementary Table S3). All publications reported that growth was in line with the expected ranges for age at 8 weeks [32,33] and 16 weeks [39], with no significant differences between groups.

## 4. Discussion

To our knowledge, this is the first systematic review to examine the effect of hypoallergenic formulae with synbiotics, in infants with IgE or non IgE mediated CMPA. It demonstrates that a specially formulated AAF with synbiotics (*Bifidobacterium breve* M16-V and prebiotics including chicory-derived oligo-fructose and long chain inulin; AAF-Syn) is as effective as AAF alone in resolving allergic symptoms and promoting normal growth, and may have additional clinical benefits for CMPA infants. Fewer infants who took AAF-Syn had infections and hospitalisations and used medications including antibiotics, compared to those on AAF. Whilst it is difficult to attribute causation, these benefits could be linked to the significant changes seen in the gut microbiota with AAF-Syn, which occurred as early as 8 weeks and were maintained up to 12 months, indicating an improvement of gut dysbiosis which is common in formula-fed infants, and those with CMPA [14,15,16,17].

While a healthy breastfed infant typically has a less diverse microbiome than an older child or adult, with a higher prevalence of *Bifidobacterium* and *Lactobacillus* [46], microbes in infants with CMPA follow an inverse pattern with a greater proportion of *Eubacterium rectale* and *Clostridium coccoides* species. This review has shown that use of AAF-Syn led to statistically significant increases in *Bifidobacterium* species, decreases in *Eubacterium rectale* and *Clostridium coccoides* species, and reduced bacterial diversity. This colonisation pattern appears to be closer to that seen in healthy breastfed infants, and could be associated in some way with the beneficial impact seen on the clinical phenotype of infants managed with this approach to treatment, with particular implications for prevention of infections.

Indeed, breastfed infants are known to be less susceptible to infections than formula fed infants. It has been hypothesised that this is due to the early colonisation of Gram-positive, anaerobic bacteria such as bifidobacteria, which promote the production of lactic and acetic acids. This results in a reduction of faecal pH which thereby inhibits the growth of some pathogens [46]. Such metabolic activity was observed in this review, with an increase in acetic acid and an associated decrease in pH from faecal samples, although findings were not consistent between studies, indicating a shortage of information in this area. Pathogen colonisation may also be inhibited by other mechanisms, as bifidobacteria compete for nutritional substrates and epithelial adhesion sites in the gut [46], which may go some way to explain the reductions in infections that were observed with AAF-Syn in this review.

There is emerging evidence to suggest that infections may be more prevalent in children with allergic conditions than their matched counterparts; in particular, the concurrence of allergy and ear infections has been extensively investigated [47,48,49,50], with studies finding an allergic origin of recurrent ear infections, particularly when IgE sensitivity is present [51,52]. Different causal mechanisms have been hypothesised, including obstruction and impaired functionality of the Eustachian tube secondary to allergic cytokine regulated inflammation [53]. While further research is required to fully understand the complex role of the gut microbiome in infective episodes, the modulating effect of gut microbiota on systemic inflammatory and immunological responses is recognised [18,46]. For example, *Bifidobacterium breve* is thought to induce epithelial cell maturation which may protect against pathogenic bacteria, and to induce anti-inflammatory processes [18]. This effect is supported by the findings of this review, in which significant changes in faecal microbial proportions among infants who received AAF-Syn was accompanied by reductions in rates of infection, including ear infections and significant reductions in hospitalisation rates.

Given the above findings, it is not surprising that the use of AAF-Syn was also associated with a reduction in the usage of medications, including antibiotics (notably amoxicillin) and in hospitalisation, which have important economic implications. Any nutritional intervention which reduces prescription of medications to treat infections is economically beneficial to health services. Each year, the net ingredient cost of medications prescribed in the community to treat infections equates to over £294 million in the UK [54,55,56,57]. Amoxicillin remains the most commonly prescribed antibiotic [58], so the reduction in its usage with AAF-Syn is of particular interest. Furthermore, the results of our simple cost analysis based on hospital admissions alone indicated potential savings per infant, which may be even greater if other changes in healthcare resources, such as specialist appointments and medications, were also taken into account.

The heterogeneity in study designs, outcomes, follow-up period and statistical assumptions make comprehensive comparison of studies in this review challenging and necessitate some caution when interpreting the results of meta-analyses. That said, the similarity of outcome data collection methods in these studies (such as fluorescent in situ hybridisation used to measure faecal microbiota) supports the use of meta-analysis for the purpose of outcome exploration, and the use of random effect models for sensitivity analysis supports our conclusions. There is also a high degree of qualitative consistency between included studies when AAF-Syn is given to infants with CMPA, showing benefits to rates of infections, antibiotic usage and hospitalisation for infection, which supports the notion of true health effects. These findings are further substantiated by other clinical trials which have demonstrated similar outcomes within different study populations, or with other infant formulae with synbiotics. A recent study of healthy formula-fed infants showed significant improvements in faecal microbiota profile, to be more similar to that of a healthy breast-fed reference group, when infants were given a whole-protein infant formula containing synbiotics for 6 weeks [59]. Another study of 115 healthy infants showed that feeding with AAF-Syn for 16 weeks led to fewer infants having infective episodes (25%) compared to AAF alone (41%) (*p*-value not given) [38]. Similarly, a study of infants with atopic dermatitis showed that fewer infants used antibiotics while receiving eHF-Syn (2.2%), compared to those who were given eHF alone (11.4%) [42]. To note, this study was not included in the review as subjects did not have established CMPA.

In fact, relatively few RCTs of hypoallergenic formulae containing synbiotics have been carried out in infants with CMPA, so whilst this review set out to summarise the published data on the use of hypoallergenic formula containing synbiotics in infants with CMPA, only a relatively small number of studies of AAF-Syn were identified and included, and all relate to a single AAF containing a specific synbiotic, effects of which are constituent-specific and should not be generalised to other pre- or probiotics. Whilst synbiotics have been combined with eHF and subjected to extensive research within cohorts of healthy infants and infants with atopic dermatitis, demonstrating similar effects on microbiota, allergic symptoms and growth within normal ranges [41,42,44], no RCTs of this eHF-Syn have been conducted in infants with CMPA. This raises interesting questions about the broader usage of hypoallergenic formulae with synbiotics, and particularly about the use of eHF-Syn in infants with diagnosed CMPA, which invites an important area for further research.

Three key findings of this review relate to the effect of AAF-Syn on gut microbiota, infections and medications. The former is measured in the predefined outcomes of the component studies, with both the latter comprised of exploratory outcomes. Whilst we consequently cannot rule out structural bias in the observations related to infections and medications, the high degree of between-study consistency would imply benefits of use.

Although the studies in this review include a range of disease severity, including both IgE and non IgE mediated allergic diagnoses, in usual clinical practice AAF are typically reserved for patients at the severe end of the allergic disease spectrum and those who do not tolerate an eHF. Given the clinical, social and financial impacts of severe CMPA, this is potentially the group of greatest clinical interest. Larger RCTs and real-world evidence studies investigating the impacts of AAF-Syn on infection rates, medication usage and hospital admissions, particularly in the longer term, would be useful to substantiate and better quantify outcomes observed in this review. In turn, a health economic analysis should be conducted to estimate the cost-effectiveness of AAF-Syn.

## 5. Conclusions

Gut dysbiosis is common in CMPA and has implications for immune and allergic development. This systematic review shows that the addition of a synbiotic to an AAF leads to allergic symptom resolution and normal growth, equally as well as AAF in infants with CMPA. In addition, the findings show that the use of AAF-Syn results in improvement of dysbiosis, and is associated with reductions in infections, medication usage (including antibiotics) and hospital admissions, with potential associated cost savings. There is a need to understand mechanisms of action, the extent to which AAF-Syn reduces infections, associated healthcare usage and the economic impacts of this, and whether similar outcomes are also achieved in infants with CMPA with eHF-Syn. Findings of this systematic review from good quality studies of heterogenous cohorts support the use of a specific AAF-Syn to improve outcomes, and the need for further research to optimise treatment protocols for infants with CMPA.

## Figures and Tables

**Table 1:** Summary of included publications.

Lead Author (Date)	Population and Type of Study	Male	Mean Age (Months)	Amount of Formula Consumed/Day (mL) Mean *± SD*	*n* AAF-Syn	*n* AAF	Intervention Duration	Timepoint Outcomes Measured
Harvey (2014) [38] Full paper	Infants with IgE mediated CMPA aged 0–36 months One arm DBPCCFC and 7 day feeding period	61%	17.3, range 3.3–46.9	Not reported	30	30	7 days	7 days
	*Full-term healthy infants aged 3–16 months, RCT* ^¶^	*67%*	*10.6, range 3–16*	*AAF-Syn: 349 ± 127*^§^; *AAF: 331 ± 124*^§^	*59*	*56*	*16 weeks*	*2, 4, 8, 12 & 16 weeks*
Burks (2015) [39] Full paper	Infants with IgE or non-IgE mediated CMPA aged 0–8 months, RCT	62%	4.5, range 0.6–8.9	Not reported. Intake was reported as comparable in both groups	54	56	16 weeks	4 & 16 weeks
Candy (2018) [32] ASSIGN study, full paper	Infants with non-IgE mediated CMPA aged 0–13 months, RCT Included breast-fed healthy reference group (not randomised)	73%	6, range 1.2–12.8	Week 8 AAF-Syn 652 ± 176; AAF 639 ± 212	35	36	8 weeks	4 & 8 weeks
Fox (2019) [33] ^†^ ASSIGN study, full paper	Infants with non-IgE mediated CMPA aged 0–13 months 26-week follow-up of Candy (2018)	73%	6, range 1.2–12.8	Week 8 AAF-Syn 652 ± 176; AAF 639 ± 212	35	36	8 weeks	8, 12 & 26 weeks
Wopereis (2019) [40] ^†^ ASSIGN study, full paper	Infants with non-IgE mediated CMPA aged 0–13 months Gene-sequencing analysis from Candy (2018) and Fox (2019)	73%	6, range 1.2–12.8	Week 8 AAF-Syn 652 ± 176; AAF 639 ± 212	35	36	8 weeks	8, 12 & 26 weeks
Chatchatee (2019) [31] PRESTO study ^‡^, conference abstract	Infants with confirmed IgE mediated CMPA aged 0–13 months, RCT	72%	9.36, SD 2.53	At 12 months: AAF-Syn: 547 ± 302; AAF: 530 ± 308	80	89	12 months	12 months
Wopereis (2020) [30] PRESTO study ^‡^, conference abstract	Infants with confirmed IgE mediated CMPA aged 0–13 months, RCT							

CMPA: cow’s milk protein allergy; RCT: randomised controlled trial; DBPCCFC: double blind placebo-controlled crossover food challenge; AAF-Syn: amino acid formula with synbiotics (Neocate Syneo^®^, Nutricia); AAF (control): amino acid formula (Neocate LCP^®^, Nutricia); ^†^ original RCT (Candy 2018 [32]) was for 8 weeks, Fox (2019) [33] and Wopereis (2019) [40] were published after original RCT; ^‡^ Chatchatee (2019) [31] and Wopereis (2020) [30] report different outcomes from same study; ^§^ Converted from reported oz/d (11.8 ± 4.3 oz/d AAF-Syn; 11.2 ± 4.2 oz/d AAF); ^¶^ Included in table for completeness, but outside scope of review as subjects were not required to have CMPA.

**Table 2:** Summary of key outcomes from included publications.

Lead Author (Date)	Population	Clinical Symptoms			Infections & Hospital Admissions	Medication Usage		Gut Microbiota	Stool Characteristics	Growth
		Allergy ^†^	GI	Resp ^‡^		Antibiotics	Other Medication ^§^			
Harvey (2014) [38]	Infants with IgE mediated CMPA aged 0–36 months	PO =								
	*Healthy infants aged 3–16 months* ^#^		✓✓		*✓*				✓✓	PO *=*
Burks (2015) [39]	Infants with IgE or non IgE mediated CMPA aged 0–8 months	=	=		✓✓	✓✓	✓✓	✓✓	✓✓	PO =
Candy (2018) [32] ASSIGN study	Infants with non-IgE mediated CMPA aged 0–13 months	=	=	=	✓	✓✓	✓	PO ✓✓	✓✓	=
Fox (2019) [33] ASSIGN study	26-week follow-up of Candy (2018)	=	=	=	✓✓		✓✓	PO ✓✓ ^¶^	=	=
Wopereis (2019) [40] ASSIGN study	Gene-sequencing analysis from Candy (2018) and Fox (2019)							PO ✓✓		
Chatchatee (2019) [31] PRESTO study	Infants with confirmed IgE mediated CMPA aged 0–13 months				✓✓					
Wopereis (2020) [30] PRESTO study	Infants with confirmed IgE mediated CMPA aged 0–13 months							PO ✓		

CMPA: cow’s milk protein allergy; GI: gastrointestinal. PO: primary outcome (PO for Chatchatee (2019) [31] was cow’s milk protein tolerance, not included in this review); ✓ numerical improvement with amino acid formula containing synbiotics (AAF-Syn) vs. amino acid formula (AAF); ✓✓ significant improvement AAF-Syn vs. AAF; = no difference; empty boxes without symbols indicate that outcome was not reported. ^†^ Atopic dermatitis/general allergy symptoms/response to double blind placebo-controlled food challenge ^‡^ Respiratory ^§^ Antifungals/emollients/functional gastrointestinal/unspecified concomitant ^¶^ Subset who received intervention for 26 weeks ^#^ Included in table for completeness, but outside scope of review as subjects were not required to have CMPA.

**Table 3:** Individual and pooled analysis of the percentage of infants who experienced infections, when receiving AAF-Syn vs. AAF.

Lead Author (Date)	AAF-Syn		AAF Alone	
	*n/N*	%	*n/N*	%
Burks (2015) [39]	1/54	1.9%	10/56	17.9%
Candy (2018) [32] ^†^ (ASSIGN study)	10/35	28.6%	12/36	33.3%
Fox (2019) [33] (ASSIGN study)	15/35	42.9%	22/35	62.9%
Chatchatee (2019) [31] (PRESTO study)	7/80 ^‡^	8.8%	18/89 ^‡^	20.2%
Pooled Result ^†^	23/169	13.6% (6.7% ^§^)	50/180	27.8% (16.3% ^§^)
Percentage reduction	51.0% (58.6% ^§^)			

AAF-Syn: amino acid formula with synbiotics. AAF: amino acid formula. ^†^ Pooled result omits data from Candy (2018) [32] as data from Fox (2019) [33] (26 week follow up) is inclusive of data from Candy (2018) [32]. ^‡^ Calculated from reported percentages of sample size. ^§^ Result weighted by study sample size.

**Table 4:** Individual publication results of the percentage of infants receiving medications with AAF-Syn vs. AAF.

Lead Author (Date)	Outcome Measures	Comparison of Findings in AAF-Syn vs. AAF Groups
Burks (2015) [39]	Systemic antibacterial and functional GI medication use included as exploratory outcome	Results AAF-Syn vs. AAF, 16-week event rates: Systemic antibacterial use: 17% vs. 34%, *p =* 0.049 - Amoxicillin use: 9% vs. 32%, *p* = 0.004 Functional GI medication use: 4% vs. 18%, *p =* 0.029
Candy (2018) [32] ASSIGN study	Systemic anti-infective & concomitant medication use included as exploratory outcome	Results AAF-Syn vs. AAF, 8-week event rates: Overall concomitant medication use: 60% vs. 78% ^†^, *p =* 0.117 - Systemic anti-infectives use: 9% vs. 33% ^†^, *p =* 0.018
Fox (2019) [33] ^‡^ ASSIGN study	Concomitant medication use included as exploratory outcome	Results AAF-Syn vs. AAF, 26-week event rates: Overall concomitant medication use: 71% vs. 83%, *p =* 0.39 - Dermatological use: 17% vs. 46%, *p =* 0.019 - Antifungal use: 0% vs. 14%, *p =* 0.054 - Emollients & protectives use: 6% vs. 29%, *p =* 0.023 - Antibiotics use ^§^: 17% vs. 31%, no *p*-value

AAF-Syn: amino acid formula with synbiotics. AAF: amino acid formula. GI: Gastrointestinal. ^†^ Percentage calculated due to error in study table. ^‡^ Data from Fox (2019) [33] is inclusive of data from Candy (2018) [32], as a 26 week follow up. ^§^ Calculated from reported size of sub-groups that did not take systemic antibiotics as a proportion of the total size of each group (Figure 1) [33].

**Table 5:** Individual publication results of changes in faecal microbiota profile among infants receiving AAF-Syn vs. AAF.

Lead Author (Date)	Outcome Measures	Comparison of Findings in AAF-Syn vs. AAF Groups	Statistical Comparison	Conclusions
Burks (2015) [39]	Secondary outcome was change in proportion of faecal BSp, CH & ER/CC	Mean AAF-Syn vs. AAF. Baseline proportions similar in both groups - BSp: 14.3% vs. 16.7%, *p =* 0.60 NS - CH: 9.3% vs. 9.1%, *p =* 0.93 NS - ER/CC: 23.8% vs. 20.1%, *p =* 0.30 NS At 16 weeks significant differences observed: - BSp: 51.2% vs. 13.0%, *p <* 0.001 - CH: 7.1% vs. 17.6%, *p <* 0.001 - ER/CC: 13.8% vs. 35.5%, *p <* 0.001	All differences between groups at 16 weeks statistically significant	“The indigenous gut microbiota of [CMPA] infants receiving an AAF can be influenced by synbiotics. As expected, synbiotics in the test formula increased *Bifidobacterium*, a genus typically predominant in the GI tract of breastfed infants” “…It can therefore be hypothesized that abolishing this gut microbiota dysbiosis may decrease [CMPA] risk or [CMPA] persistence…”
Candy (2018) [32] ASSIGN study	Primary outcome was change in proportion of faecal BSp & ER/CC Baseline measures were used as covariates for ANCOVA	Median, AAF-Syn vs. AAF. Baseline proportions not given At 8 weeks - BSp: 35.4% vs. 9.7%, *p <* 0.001 - ER/CC: 9.5% vs. 24.2%, *p <* 0.001 In the healthy breastfed comparison group: - BSp: 55.0%; - ER/CC: 6.5%	Between groups comparison for both BSp and ER/CC were statistically significant at 8 weeks	“The primary objective of modifying gut microbiota using an AAF including [synbiotics] for 8 weeks in subjects with suspected non-IgE [CMPA] was achieved.” “…The current study showed that microbial composition of infants with suspected non-IgE [CMPA] who received the test formula was closer to the profile of the HBR group than those infants receiving control formula.”
Fox (2019) [33] ASSIGN study *Subset of infants who continued intervention for 26 weeks*	26-week extension study of Candy (2018) [32]	The between-group differences in microbiota composition seen at week 8 (primary trial endpoint) were maintained with longer study follow-up. At weeks 12 and 26, the AAF-Syn group had a higher percentage of BSp and a lower percentage of ER/CC compared with the AAF group. Mean AAF-Syn vs. AAF: At baseline (0 weeks) - BSp 26.6% vs. 17.7%, *p =* 0.23 NS - ER/CC 23.6% vs. 13.4%, *p =* 0.07 NS At 8 weeks - BSp 38.4% vs. 13.1%, *p =* 0.002 - ER/CC 6.3% vs. 25.5%, *p <* 0.001 At 12 weeks - BSp 49.7% vs. 17.1%, *p =* 0.002 - ER/CC 7.5% vs. 32.1%, *p =* 0.002 At 26 weeks - BSp 48.8% vs. 15.1%, *p <* 0.001 - ER/CC 13.1% vs. 33.9%, *p <* 0.001	Between groups comparison for both BSp and ER/CC were statistically significant at 26 weeks	“…In conclusion, use of the AAF including specific synbiotics investigated in this study resulted in a sustained improvement in gut microbiota composition over 26 weeks…” “…it may suggest that the effects on gut microbiota by AAF including synbiotics can even be maintained in a [CMPA] population receiving systemic antibiotics.”
Wopereis (2019) [40] ASSIGN study	Detailed genomic characterisation of faecal microbiota, population from Candy (2018) [32] and Fox (2019) [33]. Primary outcome was the assessment of bacterial species diversity over time.	Diversity in faecal microbiota increased over time in both groups. The effect was less pronounced in the AAF-Syn group. Mean difference per week from week 0 to 26 - PD: −0.022 units, *p =* 0.069 NS - SI: −0.026 units, *p =* 0.005 Estimated average difference AAF-Syn vs. AAF was significant At 12 weeks: - PD: −0.349 units, *p =* 0.031; SI: −0.236 units, *p=* 0.049 At 26 weeks: - PD: − 0.653 units, *p =* 0.012; SI: −0.596 units, *p =* 0.002	Significant improvement in faecal microbial diversity	“…AAF including the specific synbiotics offers an effective nutritional strategy to modulate the gut microbiota of infants with suspected non-IgE mediated [CMPA] closer to a healthy breastfed profile…” “The AAF including synbiotics compared to the AAF without synbiotics showed a more gradual increment over time of bacterial diversity, which is also typically observed in longitudinal studies investigating early life gut microbiota development of breastfed infants as compared to formula-fed infants.”
Wopereis (2020) [30] PRESTO study	Detailed genomic characterisation of faecal microbiota; abundances of BSp, LSp and adult-type genera; faecal bacterial species diversity	At 6 and 12 months, compared to AAF, AAF-Syn was associated with: - Increased relative abundances of *Bifidobacterium* and *Lactobacillus* and decreased relative abundances of adult-type genera (*Blautia, Tyzzerella* and *Romboutsia*) - Lower overall bacterial diversity - Higher BSp diversity Data not given.	*p*-values not reported	“The predominant abundance of *Bifidobacterium* in subjects receiving [AAF-Syn] was reflected in lower overall diversity at 6 and 12 months.” “…Subjects receiving [AAF-Syn] showed increased diversity of species within the genus *Bifidobacterium* compared to AAF at 6 and 12 months.”

CMPA: cow’s milk protein allergy; AAF-Syn: amino acid formula with synbiotics; AAF: amino acid formula; BSp: *Bifidobacterium*; CH: *Clostridium histolyticum*; ER/CC: *Eubacterium rectale/Clostridium coccoides*; GI: Gastro-intestinal; PD: Phylogenetic diversity; SI: Shannon index; LSp: *Lactobacillus*; GI: gastrointestinal; HBR: healthy breastfed reference.

## Supplementary Materials

The following are available online at https://www.mdpi.com/2072-6643/13/3/935/s1: Table S1: Individual publication results of clinical symptoms and allergenicity in infants receiving AAF-Syn vs. AAF, Table S2: Individual publication results of stool characteristics in infants receiving AAF-Syn vs. AAF, Table S3: Individual publication results of growth parameters in infants receiving AAF-Syn vs. AAF.
