# Supplementary material for: Amino Acid Formula Containing Synbiotics in Infants with Cow’s Milk Protein Allergy: A Systematic Review and Meta-Analysis
Source: Nutrients. 2021 Mar 14;13(3):935. doi: 10.3390/nu13030935 (PMC7998621; doi:10.3390/nu13030935)
Supplement: Supplementary file 1 [file nutrients-13-00935-s001.pdf]

Supplementary Table S1. Individual publication results of clinical symptoms and allergenicity in infants receiving AAF-Syn vs AAF

Lead author (date)	Outcome measures	Comparison of findings in AAF-Syn vs AAF groups	Statistical comparison	Conclusions
Harvey (2014) [38]	Primary outcome was hypoallergenicity	All infants had negative responses to both DBPCCFC (detailed data not given).	95% confidence that at least 90% of infants and children with CMPA would have no reaction to AAF-Syn.	The results of this study demonstrate "the hypoallergenicity of [AAF-Syn] in infants and children with documented [CMPA]."
Burks (2015) [39]	Secondary outcomes included SCORAD, categorical measures of allergic symptoms and flatulence	SCORAD decreased in both groups. No significant difference between groups.<br>Allergy severity scores decreased in both groups. No difference in flatulence between groups. Detailed data not given.	Symptom outcomes described as non-significant.	"… SCORAD and allergy symptoms were not different between study groups, indicating the synbiotics-supplemented AAF is equally effective in [CMPA] management as the AAF without synbiotics when used for 16 weeks in a heterogeneous CMPA infant population…"
Candy (2018) [32]<br>ASSIGN study	Secondary outcomes included SCORAD, parent ordinal rating scales (1-4) for skin, respiratory, GI and general symptoms	All results AAF-Syn vs AAF, mean scores<br>- SCORAD change from week 0 to 8 was 12.83 to 9.63 vs 14.43 to 7.06<br>No significant differences in symptom scores reported although detailed data not presented	No p-value reported for SCORAD. Clinical symptom score changes described as non-significant.	"Overall, exploratory GI and general symptoms improved over time, but were not statistically significantly different between test and control groups at week 8."<br>Note this trial was not primarily designed to look at clinical symptoms and most participants were already receiving an eHF or AAF at inclusion, which may have offered some symptom resolution prior to the study.
Fox (2019) [33]<br>ASSIGN study	Secondary outcomes included SCORAD and parent ordinal rating scales (1-4) for skin, respiratory, GI and general symptoms	Results AAF-Syn vs AAF, median decrease in scores from week 0 to 26:<br>- SCORAD: 6.0 vs 7.0<br>Symptom scores declined to lowest possible levels by week 26 – no difference between groups.	No p-values reported	"…Likely due to use of hypoallergenic formula at baseline and associated relatively low symptoms at baseline, our analysis did not show detectable differences in clinician-assessed or parent-reported clinical symptoms between groups…"

CMPA: Cow's milk protein allergy; AAF-Syn: amino acid formula with synbiotics; AAF: amino acid formula; GI: Gastrointestinal; SCORAD: SCORing Atopic Dermatitis; eHF: extensively hydrolysed formula; DBPCCFC: double blind placebo-controlled cross-over food challenge

Supplementary Table S2. Individual publication results of stool characteristics in infants receiving AAF-Syn vs AAF

Lead author (date)	Outcome measures	Comparison of findings in AAF-Syn vs AAF groups	Statistical comparison	Conclusions
Burks (2015) [39]	Secondary outcomes included stool frequency, colour and consistency	No difference in stool frequency or consistency between groups. Post hoc analysis showed more preferred stool colour with AAF-Syn vs AAF:<br>- At week 0–2 (p = 0.014)<br>- At week 2–4 (p = 0.010)<br>- At week 4–12 (p = 0.008)<br>Detailed data not given.	Significant differences between groups in stool colour.	AAF-Syn was associated with significant differences in stool colour in infants with CMPA.
Candy (2018) [32] ASSIGN study	Secondary outcomes included stool characteristics	Mean stool frequency scores for AAF-Syn vs AAF at week 8: 1.88 ± 0.19 vs 1.98 ± 0.15, p = 0.015<br><br>No significant differences in other stool characteristics between groups (data not given)	Trend towards reduced stool frequency.	AAF-Syn was associated with significant trend towards improved stool frequency in infants with CMPA.
Fox (2019) [33] ASSIGN study	Secondary outcomes included stool characteristics	No difference in stool characteristics between groups (detailed data not given)	No p-values reported	Stool characteristics were not statistically significantly different between groups.

CMPA: cow's milk protein allergy; AAF-Syn: amino acid formula with synbiotics; AAF: amino acid formula.

*Stool consistency scale: 1 = watery, 2 = soft pudding-like, 3 = soft formed, 4 = dry formed, 5 = dry hard pellets. **Stool colour scale: 1 = green, 2 = yellow, 3 = yellow/brown, 4 = brown, 5 = black.

Supplementary Table S3. Individual publication results of growth parameters in infants receiving AAF-Syn vs AAF Growth

Lead author (date)	Outcome measures	Comparison of findings in AAF-Syn vs AAF groups	Statistical comparison	Conclusions
Burks (2015) [39]	Primary outcome was growth	Between-groups difference in Z-scores for growth parameters at 16 weeks, mean (90% CI) for AAF-Syn vs AAF:<br>- Weight: 0.147 (-0.10; 0.39) *p* = 0.32 NS<br>- Length: -0.299 (-0.69; 0.09) *p* = 0.21 NS<br>- HC: 0.152 (-0.15; 0.45) *p* = 0.40 NS<br>No difference in length for age or weight for age Z-scores	No significant difference between groups	"A specific synbiotics-supplemented AAF demonstrated normal growth and growth similar to an AAF without synbiotics when used in [CMPA] infants."
Candy (2018) [32]<br>ASSIGN study	Exploratory outcomes included growth	Growth parameters at 8 weeks were within the expected ranges for age and median Z-scores for both groups were within 1 SD of the mean (data not given)	Not reported (but see Fox below)	The synbiotic-supplemented AAF in this trial was shown to be safe in terms of…achievement of growth targets."
Fox (2019) [33]<br>ASSIGN study	Secondary outcomes included growth	Measured growth parameters were all in the expected ranges for age<br>Z-scores for growth parameters at 8 weeks, mean ± SD for AAF-Syn vs AAF:<br>- Weight for age: 0.104 ± 0.877 vs -0.137 ± 0.871<br>- Length for age: 0.570 ± 1.178 vs 0.322 ± 0.783<br>- HC for age: 0.954 ± 0.948 vs 0.495 ± 1.120, *p* =0.019<br>- Weight for length: -0.178 ± 0.840 vs -0.356 ± 1.011	Significant differences at 8 weeks for head circumference<br>No other significant differences in outcomes or at timepoints, p-values not given	"Administration of AAF with synbiotics for at least 8 weeks, and up to 26 weeks, is…associated with growth and development within the normal range."

CMPA: cow's milk protein allergy; AAF-Syn: amino acid formula with synbiotics; AAF: amino acid formula; ITT: intention to treat; NS: Not significant; HC: head circumference; SD: standard deviation
